# TUG1 long non‐coding RNA enlists the USF1 transcription factor to overexpress ROMO1 leading to hepatocellular carcinoma growth and metastasis

**DOI:** 10.1002/mco2.38

**Published:** 2020-11-26

**Authors:** Shihai Liu, Jing Qiu, Weitai He, Chao Geng, Guifang He, Changchang Liu, Duo Cai, Xiangping Liu, Ben Tian, Huazheng Pan

**Affiliations:** ^1^ Medical Animal Laboratory The Affiliated Hospital of Qingdao University Qingdao China; ^2^ Department of stomatology Qingdao Municipal Hospital Qingdao China; ^3^ School of Biological Science and Technology University of Jinan Jinan China; ^4^ Department of Clinical Laboratory The Affiliated Hospital of Qingdao University Qingdao China; ^5^ Medical Research Center The Affiliated Hospital of Qingdao University Qingdao China; ^6^ Department of Neurosurgery Intensive Medicine The First Affiliated Hospital of Baotou Medical College Baotou China

**Keywords:** hepatocellular carcinoma, long non‐coding RNA, metastasis, taurine up‐regulated gene 1

## Abstract

Hepatocellular carcinoma (HCC) is a prevalent and highly aggressive cancer. Long non‐coding RNAs (lncRNAs) are recognized as potential molecular targets for HCC and are currently under increased research focus. Here, we investigate the regulatory processes underlying the axis of the lncRNA taurine upregulated gene 1 (TUG1), Upstream Transcription Factor 1 (USF1), and reactive oxygen species modulator 1 (ROMO1) in the propagation and metastasis of HCC cells. Distribution of lncRNA TUG1 was found to be prominent in HCC cell cytoplasm and nuclei. LncRNA TUG1 conscripted the USF1 transcription factor to enhance the promoter function of ROMO1. Enlisting the USF1 transcription factor to increase ROMO1 expression following upregulation of TUG1 lncRNA enhanced HCC Huh7 cell proliferation, motility, and metastasis. Rapid tumor proliferation in nude mice provided in vivo verification. The importance of the lncRNA TUG1/USF1/ROMO1 complex as a target for HCC therapy is a key result of this investigation which is exemplified by its role in regulating the proliferation, motility, and metastasis of HCC cells.

## Graphical abstract

Distribution of LncRNA TUG1 was prominent in HCC cell cytoplasm and nuclei. LncRNA TUG1 conscripted the USF1 transcription factor to enhance the promoter function of ROMO1. Enlisting the USF1 transcription factor to increase ROMO1 expression following upregulation of TUG1 lncRNA enhanced HCC cell proliferation, migration and metastasis.

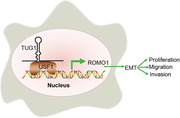

**Abbreviations**
ChIP: chromatin immunoprecipitation
DAPI: 4′,6‐diamidino‐2‐phenylindole
ECM: extracellular matrix
EdU: 5‐ethynyl‐2′‐deoxyuridine
FBS: fetal bovine serum
FISH: fluorescence in situ hybridization
GAPDH: glyceraldehyde3‐phosphate dehydrogenase
GEO: gene expression omnibus
HCC: hepatocellular carcinoma
HRP: horseradish peroxidase
lncRNAs: long noncoding RNAs
MMP‐2: matrix metalloproteinase‐2
NCCN: national comprehensive cancer network
OE‐NC: negative control of overexpression vector
PBS: phosphate buffered saline
PVDF: polyvinylidene fluoride
qRT‐PCR: quantitative real‐time polymerase chain reaction
RIP: RNA immunoprecipitation
RLU: relative luciferase unit
ROMO1: recombinant reactive oxygen species modulator 1
SDS: sodium dodecyl sulphate
SDS‐PAGE: sodium dodecyl sulphate‐polyacrylamide gel electrophoresis
sh‐NC: negative control of shRNA
TGF‐β1: transforming growth factor β1
TUG1: taurine upregulated1
USF1: upstream stimulatory factor 1

## 1 BACKGROUND

Globally, over 850 000 additional patients are diagnosed annually with liver cancer, making it the second leading cause of worldwide cancer mortality.^1^, ^2^ Nearly 90% of these patients have hepatocellular carcinoma (HCC) which is the most prevalent form of primary liver cancer.^3^ The number of new cases and deaths from HCC in China is well above the world average.^4^, ^5^, ^6^ The frequency of HCC recurrence and its high metastatic capacity have restricted the 5‐year survival rate despite developments in HCC therapy over the last few decades.^7^ Invasive metastases are a typical cause of high mortality in cancer patients.^8^ The latest medical data indicate that, in addition to being a considerable obstacle to treatment, incipient or recurrent metastases from primary or secondary cancers kill as many as 90% of cancer patients.^9^ New research has aimed to discover novel biochemical targets to enhance the diagnosis and long‐term prognosis of cancer patients as well as to provide information to guide medical decision making.^10^

Studies based on transcriptomics and genomics show that approximately 75% of human DNA transcribed to RNA is not translated into protein.^11^, ^12^ Many such RNA sequences exceed a length of 200 nucleotides and are termed long non‐coding RNAs (lncRNAs). Several investigative reports applying various experimental techniques have demonstrated that these lncRNAs influence many essential physiological functions, including the differentiation of cell lineages^13^, ^14^ and immunological reactions.^15^ Moreover, lncRNAs may function inappropriately in cancer and other diseases, suggesting that they may have pathological roles as well.^16^, ^17^ Indeed, proof of lncRNA involvement in cancer symptomology has recently surfaced.^18^ In terms of HCC metastases, little is known of the physiological roles and biochemical pathways mediated by lncRNAs, suggesting the potential usefulness of their characterization.

The progression of HCC has recently been shown to be highly dependent on lncRNAs.^19^, ^20^ This is consistent with findings demonstrating an association between abnormal cell activity leading to malignancy and mutated lncRNA sequences.^21^, ^22^ Notably, the lncRNA taurine up‐regulated1 (TUG1) was shown to promote cell death while its knockdown enhanced cellular survival.^23^ Increased HCC invasion was also found to correlate with raised levels of aberrant reactive oxygen species modulator 1 (ROMO1).^24^ Another potential HCC treatment target is the oncogenic upstream transcription factor 1 (USF1) which has recently been shown to contribute significantly to HCC proliferation.^25^ Considering the above in combination, we hypothesized that the lncRNA TUG1/USF1/ROMO1 complex may have a pathological role in HCC. We thus investigated TUG1 to clarify its potential for an HCC therapeutic approach.

## 2 MATERIALS AND METHODS

### 2.1 Ethics statement

The Ethics Committee of the Hospital of Qingdao University gave us permission to carry out our research. Each patient that took part provided written informed consent. The Laboratory Animal Care and Use Committee of the Qingdao University Affiliated Hospital (AHQU20180822A) approved the design and conduct of all our in vivo animal experiments.

### 2.2 Tissue samples and cell culture

Included in the study were samples post‐surgically retrieved from 120 HCC patients in the period commencing January 2010 and ending October 2014 at the Hospital of Qingdao University. None of the patients had received radiation or chemotherapy before the surgery. After surgical harvesting, the tumors were rapidly frozen, categorized following the World Health Organization's recommendations for liver cancer classification, and stored until required for examination. The National Comprehensive Cancer Network's guidelines were followed in treating study participants with HCC. Careful monitoring of each patient continued post‐study, and overall survival (OS) was typically measured from the surgery date until death. The presence of the following exclusion criteria was considered for all patients before enrollment: (1) insufficient post‐study medical records; (2) metastases outside the liver; (3) death within the first 10 days due to the surgical operation; and (4) HCC recurrence. Additionally, patients were included if the following were met: (1) A or B Child‐Pugh liver disease classification; (2) absence of other cancers; and (3) no radiofrequency ablation before surgery. A list of the clinical characteristics of patients is given in Table 1.

**TABLE 1:** Correlation between TUG1 expression and clinicopathologic characteristics of HCC patients

Characteristics TUG1	TUG1 expression		
	Low no. of cases (%)	High no. of cases (%)	*P* value
**Age**			
<50	15 (12.5)	10 (8.3)	.261
≥50	45 (37.5)	50 (41.7)	
**Gender**			
Male	49 (40.8)	50 (41.7)	.810
Female	11 (9.2)	10 (8.3)	
**Alcoholism**			
Yes	15 (12.5)	20 (16.7)	.315
No	45 (37.5)	40 (33.3)	
**Liver cirrhosis**			
Yes	30 (25.0)	41 (34.2)	**.041**
No	30 (25.0)	19 (15.8)	
**AFP (ng/L)**			
<200	46 (38.3)	44 (36.7)	.673
≥200	14 (11.7)	16 (13.3)	
**ALT (U/L)**			
<60	44 (36.7)	40 (33.3)	.426
≥60	16 (13.3)	20 (16.7)	
**AST (U/L)**			
<40	45 (37.5)	35 (29.2)	.053
≥40	15 (12.5)	25 (20.8)	
**Tumor number**			
Single	46 (38.3)	32 (26.7)	**.007**
Multiple	14 (11.7)	28 (23.3)	
**Tumor size**			
<5 cm	39 (32.5)	34 (28.3)	.350
≥5 cm	21 (17.5)	26 (21.7)	
**Portal vein invasion**			
Yes	14 (11.7)	12 (10.0)	.658
No	46 (38.3)	48 (40.0)	
**TNM stage**			
I+II stage	56 (46.7)	50 (41.7)	.088
III+IV stage	4 (3.3)	10 (8.3)	

*P* values are calculated using chi‐square test. Bold values indicate significant differences (*P *< .05).

Abbreviations: AFP, α‐fetoprotein; ALT, alanine aminotransferase; AST, aspartate aminotransferase; HCC, hepatocellular carcinoma; TNM, tumor, node, metastasis.

Shanghai Institutes for Biological Sciences (Chinese Academy of Sciences, China) supplied HepG2, SK‐HEP1, L02, PLC/PRF5, Huh7, and Hep3B cell lines, and the American Type Culture Collection (Manassas, VA) provided the SNU398 cells. We cultured the cells in RPMI 1640 medium containing penicillin (100 U/mL), streptomycin (100 μg/mL), and fetal bovine serum (FBS, 10%) in an incubator with 5% CO_2_ set at 37°C. When cells were 80% confluent, they were considered ready for passaging.

### 2.3 Transfection of HCC cells

Non‐silencing shRNA negative control (sh‐NC) + an empty vector for overexpression (OE) were used as an NC (OE‐NC); OE‐NC alone, sh‐NC alone, sh‐NC + a vector overexpressing TUG1 (OE‐TUG1), sh‐TUG1 + OE‐NC, OE‐TUG1 alone, sh‐TUG1, OE‐TUG1 + sh‐ROMO1, OE‐TUG1 + sh‐USF1, OE‐TUG1 + sh‐NC + OE‐USF1, and OE‐TUG1 + OE‐USF1 + sh‐ROMO1 were used to transfect HCC cells. GeneChem Corporation (Shanghai, China) synthesized the above plasmid vectors. Cells were cultured in six‐well plates for 2 days until approximately 80% confluent and transfected over 8 hours using the lipofectamine 3000 reagent (Thermo Fisher Medical, MA) Transfection was terminated by media replacement, and gene expression or silencing was left to proceed for 2 days before cells were examined further.

### 2.4 Quantitative real‐time polymerase chain reaction gene expression measurement

The RNAiso Plus reagent (TaKaRa, Kyoto, Japan) was used, according to the manufacturer's instructions, for extraction of total RNA from the cells the total cellular RNA was transcribed back into cDNA using the TaKaRa reverse transcription assay, and the SYBR Premix EX Taq package (TaKaRa) was used for quantitative real‐time polymerase chain reaction (qRT‐PCR). A LightCycler480 (Roche Diagnostics, Germany) fitted with a SYBR Green I filter (483 nm) was used to carry out qRT‐PCR. Sangon Biotech Co., Ltd., (Shanghai, China) constructed the primers, and their sequences are provided in Table S1. Levels of mRNA were normalized to glyceraldehyde 3‐phosphate dehydrogenase (GAPDH), and amplification efficiency was derived via the **2** ^−ΔΔCt^ approach.^26^

### 2.5 Cell proliferation assays

The 5‐ethynyl‐2′‐deoxyuridine (EdU) and Cell Counting Kit‐8 (CCK‐8; Dojindo Laboratories, Kumamoto, Japan) incorporation assays were used to determine cell proliferation. Identical quantities of indicated osteosarcoma cells were put in each well of a 96‐well plate for CCK‐8 assay. Following the manufacturer's instructions to carry out the CCK‐8 assay, we recorded the 450 nm absorbance every 24 hours.

Cells were plated at 1 × 10^6^ cells/mL in EdU solution (C0075S; Beyotime, Jiangsu, China), incubated at room temperature for 2 hours, and washed with phosphate‐buffered saline (PBS). Polyoxymethylene (40 g/L) was then used to fix the cells for half an hour at room temperature. The cells were then washed with PBS followed by another wash with PBS supplemented with Triton X‐100 (0.5%). Following the addition of Azide 555 staining solution (C0075S; Beyotime, Jiangsu, China), cells were left in the dark for half an hour at room temperature and were then examined under a fluorescence microscope (at a magnification of × 100). We counted proliferating cells that stained positively with 4′,6‐diamidino‐2‐phenylindole (DAPI) and EdU. The cell proliferation rate was derived by dividing the number of positively‐stained proliferating cells by all available cells and multiplying the result by 100 to give a percentage. Three separate runs of this process were performed to give triplicate results.

### 2.6 Fluorescence in situ hybridization assay

The lncRNA TUG1 RNA probes were labeled with Fluorescein Isothiocyanate (FITC) and added to fix Huh7 cells. Gene Pharma (Shanghai, China) provided the FISH assay and their protocol was followed for all Fluorescence in situ hybridization (FISH) experiments. In brief, Huh7 cells were cultured in 48‐well plates, and adherent cells were rinsed with PBS before fixation with paraformaldehyde (4%) for 15 minutes at room temperature. Cells were then incubated for 15 minutes with Triton X‐100 (0.1%) to allow permeation of the FITC‐bound probes which were then allowed to hybridize overnight in the dark. This was then followed by 5 minutes of washing at 42°C with formamide and Tween 20 (0.1%), and the cells were examined under a fluorescence microscope to observe nuclei stained with DAPI and to allow the monitoring of lncRNA TUG1 placement inside the cells.

### 2.7 RNA immunoprecipitation

Binding off lncRNA TUG1 to the USF1 transcription factor was investigated with the RNA immunoprecipitation (RIP) kit (Millipore, MA). Cells were pretreated with ice‐cold lysis buffer, centrifuged, and the supernatants harvested. Anti‐USF1 antibodies or negative control IgG (Abcam, Cambridge, MA; diluted 1:100) conjugated to magnetic beads in RIP Wash Buffer were then added to the supernatant to co‐precipitate RNA at 4°C overnight. Antibody‐conjugated beads with bound RNA were isolated, treated with proteinase K to digest proteins, and the RNA retained for further examination via PCR.

### 2.8 Western blotting assay

The total cell or tissue proteins were isolated by incubation with a proteinase inhibitor cocktail (Sigma, St Louis, MS) in RIPA lysis buffer (PBS containing phenylmethylsulphonyl fluoride (100 μg/mL), NP40 (1%), sodium dodecyl sulfate (SDS, 0.1%), sodium deoxycholate (0.5%)) for 30 minutes on ice. The lysates were centrifuged, the supernatants were collected, and the proteins were separated by SDS‐polyacrylamide gel electrophoresis. Transfer of the electrophoresed proteins to polyvinylidene fluoride membranes was followed by membrane blocking for 1 hour at room temperature using a skimmed milk solution (5%). The following antibodies (all diluted 1:1 000) were then used to probe the membranes at 4°C overnight: mouse anti‐E‐cadherin (Cell Signaling Technology, Danvers, MA), rabbit anti‐SNAIL (Cell Signaling Technology), mouse anti‐ROMO1 (Abcam, Cambridge, MA), rabbit anti‐N‐cadherin (Cell Signaling Technology), rabbit anti‐matrix metalloproteinase‐2 (MMP‐2) (Cell Signaling Technology), rabbit anti‐USF1 (Abcam), mouse anti‐USF1 (Santa‐Cruz Biotechnology, Santa Cruz, CA), and mouse anti‐ROMO1 (Abcam). Additionally, rabbit anti‐GAPDH (Cell Signaling Technology) was used as a loading control at a dilution of 1:3000. Membranes were incubated with 1:2000 dilutions of either anti‐mouse H&L IgG secondary antibody (Abcam) or anti‐rabbit anti‐goat H&L IgG secondary antibody (Abcam), followed by incubation with horseradish peroxidase for 1 hour, and visualization of the protein bands with Clarity Western ECL Blotting Substrates (Bio‐Rad, CA). Bio‐Rad's ChemiDoc XRS+ Imaging System allowed the capturing of the developed images.

### 2.9 Dual luciferase reporter gene assay

The ROMO1‐2Kb luciferase reporter plasmid was transfected into Huh7 cells together with sh‐NC, OE‐NC, sh‐TUG1, and OE‐TUG1 to investigate the impact of lncRNA TUG1 on ROMO1 promoter function. Upon transfection, cells were extracted and lysed for 48 hours. A luciferase gene assay was carried out using a luciferase assay system from Promega (Madison, WI) based on a dual luciferase gene analysis method. Renilla luciferase was used for comparison. The extent of target reporter gene activation was calculated as the ratio of firefly luciferase to Renilla luciferase units. The JASPAR database of transcription factor binding profiles (www.jasper.genereg.net) and the University of California Santa Cruz (UCSC) Genome Browser (www.genome.ucsc.edu) web‐based repositories facilitated bioinformatics analysis, and estimated that two ROMO1 promoter sites were bound by the USF1 protein. A luciferase reporter plasmid carrying an aberrant or specific adhesion domain was transfected into Huh7 cells together with the vector expressing USF1 to check the unique site of USF1 adhesion to DNA coding for the ROMO1 promoter. Binding was then monitored by assessing the gene assay with a luciferase reporter.

### 2.10 Chromatin immunoprecipitation

To induce the development of DNA‐protein cross‐linking, Huh7 cells were fixed with formaldehyde for 10 minutes. Then, we used a sonicator for separation of the chromatin. The harvested cells were split into two containers that were separately incubated with an anti‐USF1 antibody (Santa‐Cruz Biotechnology) and NC rabbit anti‐IgG antibody (Abcam) at 4°C overnight. After centrifugation for 10 minutes at 12 000 g at 4°C, protein sepharose/agarose was used to precipitate the protein‐DNA conjugates which were centrifuged again at 12 000 g for 5 minutes, and the supernatant discarded. The heterogenous conjugates were isolated, and cross‐linkages were reversed by incubation overnight at 65°C. Chloroform/phenol purification of the harvested DNA prepared samples for PCR. One set of primers ensured amplification of the ROMO1 promoter region binding USF1 (site 1). An additional set of primers ensured amplification of the DNA sequence beyond the ROMO1 promoter DNA and served as a negative control. Both sets of primers together were used for qRT‐PCR to confirm the ROMO1 DNA sequence binding USF1. A chromatin immunoprecipitation (ChIP) assay was carried out with the purified DNA fragment following lncRNA TUG1 silencing. In other control experiments, sh‐NC was used.

### 2.11 Transwell assays

Serum‐free water was used to dilute extracellular matrix (ECM) Matrigel to 1 mg/mL. A 24‐well Transwell upper chamber was coated with ECM on its polycarbonate layer, and then incubated in 5% CO_2_ for 5 hours at 37°C to promote its hardening. The chamber was incubated with Roswell Park Memorial Institute (RPMI) 1640 for 30 minutes at 37°C for rehydration of the Matrigel after all unnecessary liquid had been drained. This step was not carried out in the migration test. Huh7 cells were starved of serum for 24 hours, removed, and centrifuged. Cells were resuspended at 2 × 10^5^ cells/mL in serum‐free RPMI 1640. Then the bottom chamber was filled with 0.7 mL of medium with FBS (10%) while 0.2 mL of cells were added to the top chamber. The cells were grown in CO_2_ (5%) for 24 hours at 37°C under humid conditions. The cells on the membrane that did not migrate were removed together with those in the chamber. Migrated, adherent cells were methanol‐fixed for half an hour and stained for 20 minutes with crystal violet (0.2%). An inverted microscope was used to view and photograph the cells. Lastly, the number of cells from five random view fields was recorded.

### 2.12 Xenograft in nude mice

Vitalriver (Beijing, China) supplied thirty female BALA/C nude mice (17‐20 g weight, aged 4‐6 weeks) that were kept at 45‐50% humidity and 26 ± 1°C temperature. We randomly separated the mice into five groups of six mice each for the following experiments: OE‐TUG1+OE‐USF1+sh‐ROMO1, sh‐NC alone, OE‐TUG1+OE‐USF1+sh‐NC, sh‐TUG1 alone, and OE‐NC+sh‐NC. Cells were cultured after transfection with the lentivirus vectors until they were approximately 80% confluent, then they were harvested, pelleted by centrifugation, and suspended at a concentration of 1 × 10^7^ cells/mL in media. Thigh tissue from the nude mice was covered with the cell suspension (100 μl) allowing tumor development for subsequent detection and imaging. When the implanted cells were visible, Vernier calipers were used to periodically record the volume ([width × length × width]/2) of the growing tumor. The mice were sacrificed after 23 ± 1 days. The tumor growth curves were constructed after harvesting of the tumors from the dead mice and the tumor weights determined. Formalin (10%) was used to fix the mouse tissues allowing the detection of tumors and hepatic metastases by paraffin embedding and sectioning into 3 μm slices. H&E staining was performed using a standard procedure.

### 2.13 Statistical analysis

The Statistical Analysis Software Package (SPSS) Version 18.0 (IBM Corp., Armonk, NY) was used to carry out all statistical tests. The chi‐square test enabled the assessment of correlations between patients' symptoms and expression. Separately, the Spearman's rank correlation coefficient allowed bivariate associations among lncRNA TUG1 research variables to be determined. Kaplan‐Meier survival curves were constructed, and the log‐rank test was used to measure differences. Data are indicated as mean ± SD. A probability (*P*) value of *P *< .05 was taken as the statistical significance threshold, and data are represented as mean ± standard deviation.

## 3 RESULTS

### 3.1 High lncRNA TUG1 HCC expression correlates with poor prognostic outcomes

**FIGURE 1:**
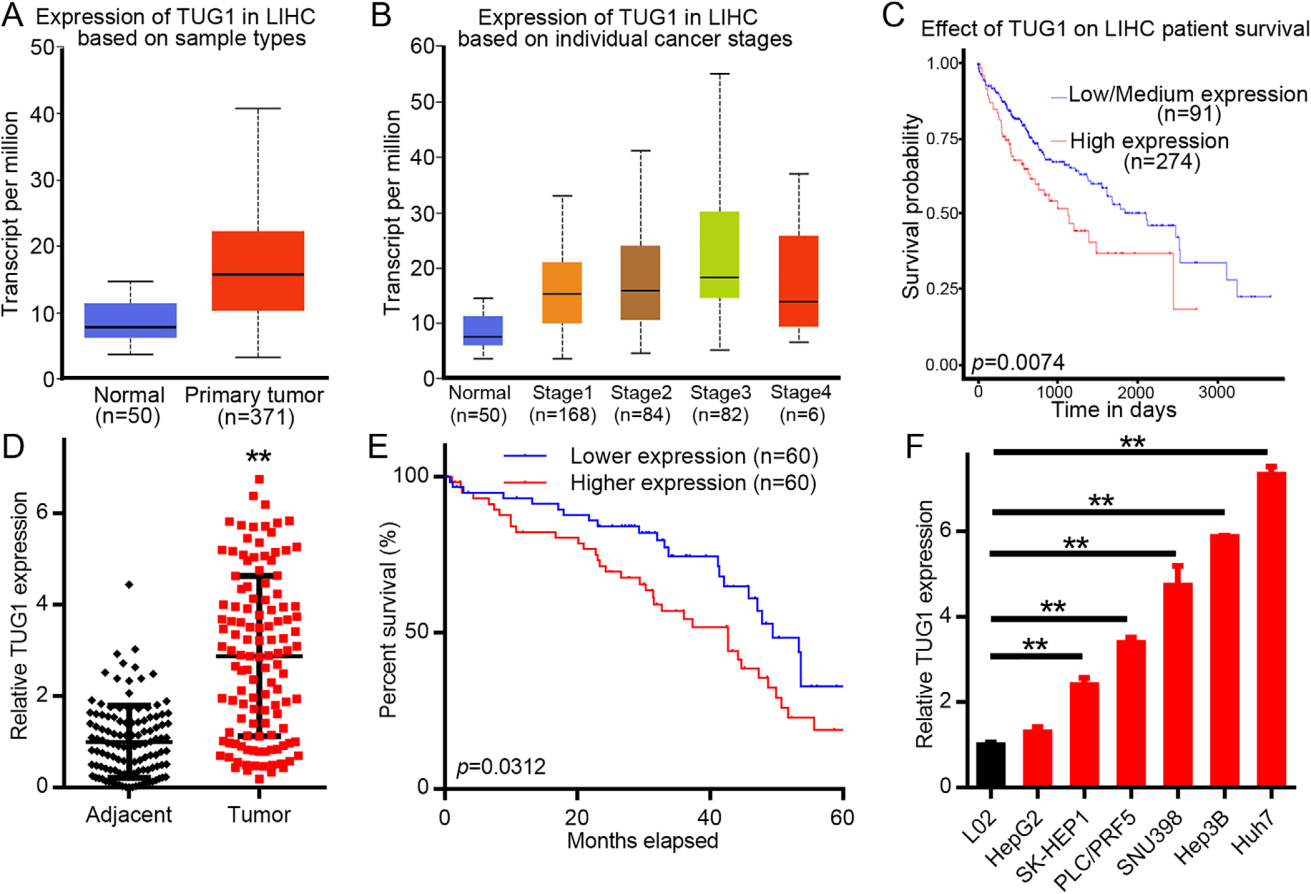
Poor HCC patient prognosis correlates with lncRNA TUG1 which is strongly expressed in HCC. A, TCGA database analysis using UALCAN datasets for the expression of lncRNA TUG1. B, The expression of lncRNA TUG1 mRNA in primary HCC tissues from the UALCAN online resource in patients of different clinical stages. C, HCC patient prognosis correlates with UALCAN study of lncRNA TUG1. D, Presence of lncRNA TUG1 in the HCC tissues (n = 120) and neighboring liver tissues (n = 120) was tested by qRT‐PCR, **P < .01. E, Examination of the correlation between HCC patient prognosis and lncRNA TUG1 expression using the Kaplan‐Meier survival assessment. F, Investigation of lncRNA TUG1 expression in six HCC cell types: PLC/PRF5, SK‐HEP1, HepG2, SNU398, Huh7, Hep3B and one non‐cancerous liver cells L02, **P < .01. The cell assays were repeated separately three times. Results are shown as mean ± standard deviation. Differences between two groups were assessed using the paired *t*‐test and those between multiple groups were compared using Tukey's post hoc test following one‐way ANOVA. Hospital survival rate was measured using the Kaplan‐Meier approach and for univariate analysis and log‐rank testing was carried out

Using UALCAN for differential expression analysis of the TCGA gene expression dataset showed a high degree of expression of lncRNA TUG1 in HCC tissues (Figure 1A). Furthermore, Figure 1B illustrates that findings from studying the public data showed that differences in expression rates of lncRNA TUG1 between clinical stages were easily distinguishable, with substantially higher expression in advanced stage HCC patients. In addition, observations from the publicly available online RNA sequence‐based tool, Kaplan‐Meier Plotter RNA, revealed that increased amounts of lncRNA TUG1 were closely correlated with lower HCC OS (Figure 1C).

Table 1 summarizes lncRNA TUG1 expression in 120 stored human HCC tumors, comprising 106 at stages I and II together with 14 at stages III and IV that were tested to verify the bioinformatics results. We used standard scoring for qRT‐PCR (Table S1) and found that TUG1 was more highly expressed in HCC tumors than in normal liver tissue (Figure 1D). High TUG1‐expressing patients were shown to have a significantly lower OS than patients with low‐level expression, demonstrated by Kaplan‐Meier survival curves (Figure 1E).

The expression of lncRNA TUG1 was then investigated by qRT‐PCR in six HCC cell lines, HepG2, SK‐HEP1, PLC / PRF5, SNU398, Hep3B, Huh7, and the non‐cancerous L02 (Figure 1F). The six HCC cell lines showed significantly higher lncRNA TUG1 expression compared with L02. The Huh7 cell line was used for further study as it was the cell type with the highest expression. Considered in combination, these results revealed abnormal expression of lncRNA TUG1 in HCC which correlates with patient survival.

We also studied the relationship of HCC symptomology with TUG1. Significant correlations of TUG1 expression with the number of tumors (*P *= .007) and liver cirrhosis (*P *= .041) were seen (Table 1). However, gender (*P *= .810), aspartate transaminase (*P *= .053), age (*P *= .261), alcoholism (*P *= .315), alpha‐fetoprotein (*P *= .673), alanine transaminase (ALT) (*P *= .426), tumor scale (*P *= .350), portal vein invasion (*P *= .658), and TNM stage (*P *= .088) were not correlated with the expression of TUG1. For HCC prognosis, we analyzed the dangers posed by TUG1. Cox‐regression analysis was used to decide whether TUG1 would function as a risk factor. Table 2 demonstrates the Cox regression univariate analysis results that indicated how elevated TUG1 expression correlated with a substantially raised likelihood of death in HCC patients (*P *< .05). In the multivariate analysis, TUG1 and AST level were included into the model (Table 2). Multivariate Cox regression analysis also showed that TUG1 could forecast a decreased likelihood of survival (*P *= .044), as could the AST level (*P *= .002) (Table 2). These findings show a strong association between HCC prognosis and TUG1 expression.

**TABLE 2:** Univariate and multivariate analyses of various prognostic parameters in patients with HCC Cox‐regression analysis for OS

	Univariate analysis			Multivariate analysis		
	*P* value	Hazard Ratio	95% CI	*P* value	Hazard Ratio	95% CI
**TUG1**	**.034**	1.763	1.044‐2.977	**.044**	1.713	1.013‐2.895
**AST** (U/L, < 40 vs. ≥40)	**.002**	2.331	1.370‐3.966	**.002**	2.277	1.338‐3.877
Age (years, < 50 vs. ≥50)	.068	2.087	0.947‐4.600			
Gender (male/female)	.778	0.907	0.459‐1.793			
Alcoholism (yes/no)	.482	1.237	0.684‐2.238			
Liver cirrhosis (yes/no)	.728	0.912	0.541‐1.536			
AFP (ng/L, < 200 vs. ≥200)	.904	1.037	0.576‐1.867			
ALT (U/L, < 60 vs. ≥60)	.082	1.621	0.940‐2.793			
Tumor number (single/multiple)	.916	0.971	0.569‐1.659			
Tumor size (cm, < 5 vs. ≥5)	.569	0.857	0.504‐1.458			
Portal vein invasion (yes/no)	.276	0.660	0.312‐1.395			
TNM stage (I+II vs III+IV)	.460	0.876	0.617‐1.244			

Bold values indicate significant differences (*P* < .05).

Abbreviations: AFP, α‐fetoprotein; ALT, alanine aminotransferase; AST, aspartate aminotransferase; HCC, hepatocellular carcinoma; OS, overall survival; TNM, tumor, node, metastasis; 95% CI, 95% confidence interval.

### 3.2 Inhibition of invasion and migration, and induction of HCC EMT by silencing lncRNA TUG1

**FIGURE 2:**
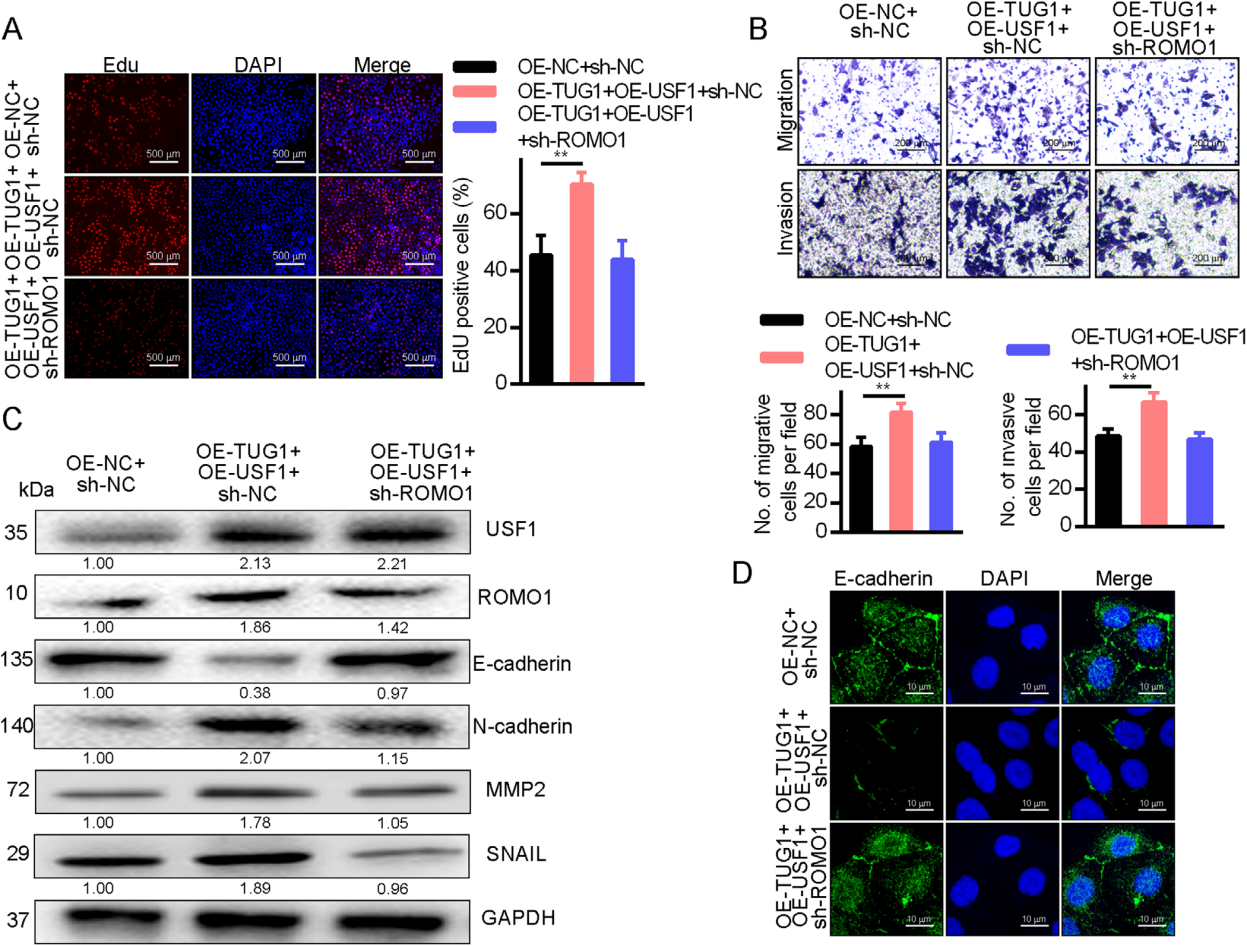
LncRNA TUG1 silencing prevents motility and metastasis, and causes HCC cell EMT. A, qRT‐PCR evaluated the extent of lncRNA TUG1 upregulation or silencing in Huh7 cells, **P < .01. B, CCK‐8 cell proliferation assay after OE‐TUG1 or sh‐TUG1 transfection, *P < .05, **P < .01. C, Images of the proliferation of HCC cells transfected by EdU staining with OE‐TUG1 or sh‐TUG1, **P < .01. D, Huh7 cell capacity for motility and metastasis measured by a Transwell assay (×100), *P < .05, **P < .01. E, Levels of SNAIL, N‐cadherin, E‐cadherin, and MMP‐2 in Huh7 cells measured by Western blotting using GAPDH as the loading control. F, The levels of E‐cadherin protein after OE‐TUG1 or sh‐TUG1 transfection were identified by immunofluorescence. Data are shown as mean ± standard deviation. Variations between different cohorts were assessed using Tukey's post hoc test following one‐way ANOVA. Assays were performed separately in triplicate

The influence of lncRNA TUG1 on the physiological properties of HCC cells was investigated using TUG1 silencing or overexpression. The significant level of Huh7 cells lncRNA TUG1 silencing or over‐expression satisfied the criteria for further studies (Figure 2A). EdU and CCK‐8‐staining experiments revealed that enhanced expression of TUG1 greatly increased cell proliferation, and lncRNA TUG1 silencing reversed this condition (Figure 2B,2C). Figure 2D shows that Huh7 cell invasion and migration were increased by lncRNA TUG1 upregulation, while this was prevented by lncRNA TUG1 silencing, as demonstrated by the Transwell assay. Incubation with sh‐NC+OE‐TUG1 increased expression of the interstitial markers SNAIL, N‐cadherin, and MMP‐2 and decreased that of the epithelial marker E‐cadherin relative to sh‐NC+OE‐NC exposure as shown by Western blotting (Figure 2E). In tandem, sh‐TUG1+OE‐NC incubation, relative to that with sh‐NC+OE‐NC, resulted in increased E‐cadherin expression, with decreased SNAIL, MMP‐2, and N‐cadherin expression. Using immunofluorescence, we observed similar findings for E‐cadherin in Huh7 cells (Figure 2F). Taken together, these findings suggest that lncRNA TUG1 silencing ameliorates growth, recruitment, and metastasis of HCC cells.

### 3.3 ROMO1 gene expression was enhanced by lncRNA TUG1 through enlisting the USF1 transcription factor

**FIGURE 3:**
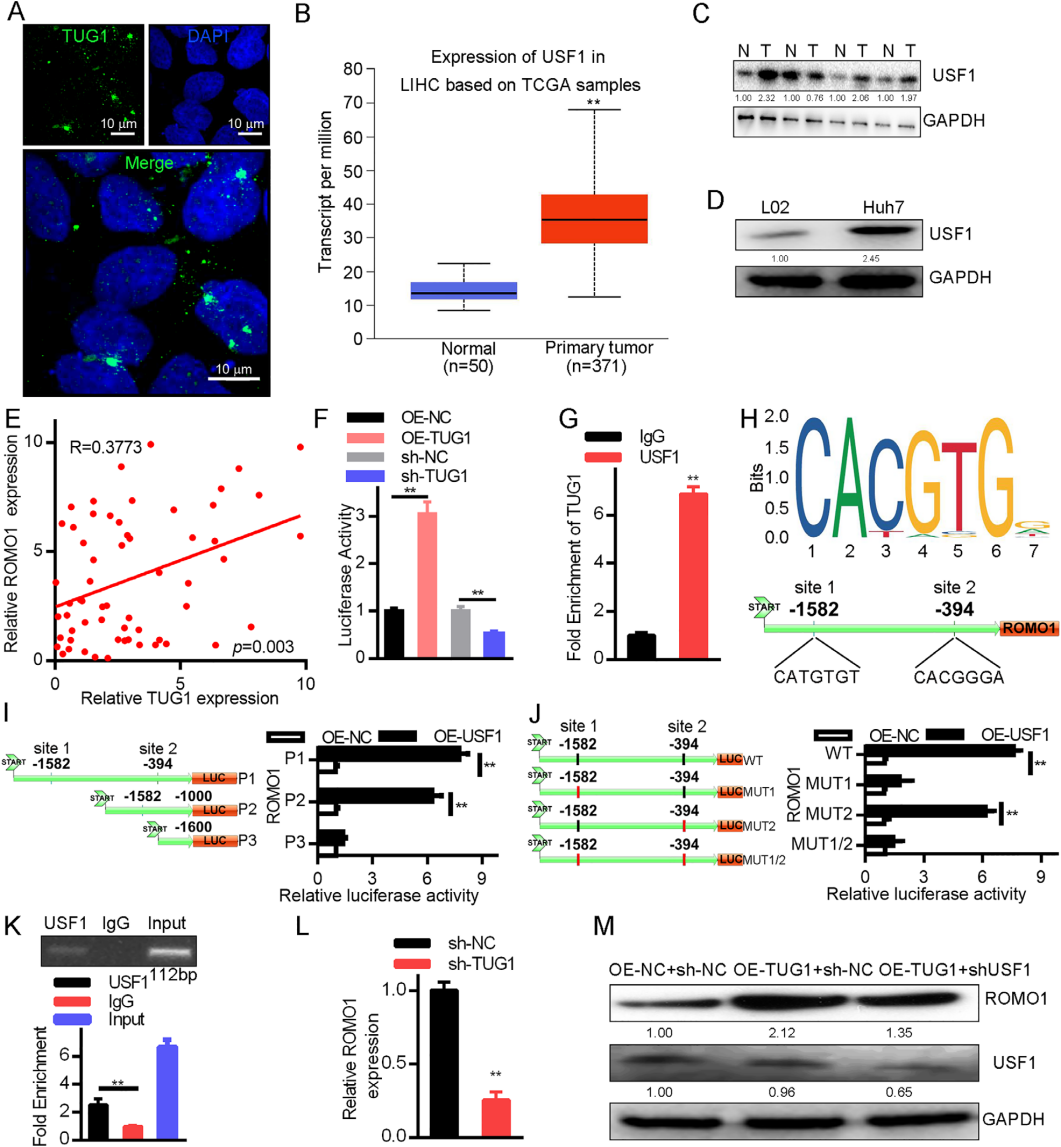
ROMO1 expression is regulated by lncRNA TUG1 by mobilizing the USF1 transcription factor. A, Subcellular position of lncRNA TUG1 in Huh7 (×630) cells measured by RNA‐FISH. B, HCC correlated with USF1 expression in the UALCAN microarray dataset, **P < .01. C, GAPDH was used to standardize HCC tissue USF1 expression in Western blotting experiments. D, GAPDH was used to standardize Huh7 cells USF1 expression in Western blotting experiments. E, HCC tissue correlations between ROMO1 and lncRNA TUG1 were assessed using Spearman's correlation (n = 60). F, Impact of lncRNA TUG1 on ROMO1 promoter function was examined using the dual‐luciferase reporter gene assay, **P < .01. G, RIP assay confirmed that USF1 interacted with lncRNA TUG1, **P < .01. H, ROMO1 DNA promoter sites most likely to bind to the USF1 transcription factor. I, Expression vector for USF1 and recombinant luciferase reporter vector for ROMO1 constructed for Huh7 cells transfection in the reporter gene assay with dual‐luciferase, **P < .01. J, For the dual‐luciferase reporter gene assay, the USF1 and ROMO1 vectors were transfected into Huh7 cells, **P < .01. K, ROMO1 promoter region binding site 1 was tested using the ChIP assay for USF1 binding capacity relative to IgG, **P < .01. L, ROMO1 level modulation by raised USF1 was monitored by the ChIP assay following lncRNA TUG1 silencing in Huh7 cells, **P < .01. M, GAPDH was used to standardize USF1 and ROMO1 in each group during Western blotting experiments. Data are shown as mean ± standard deviation. Using a paired or unpaired *t*‐test as required, parametric results between two paired or unpaired groups, respectively, were carried out. Tukey's post hoc test following one‐way ANOVA compared differences among more than two groups. Three independent experimental replicates were performed

FISH showed specific compartmentalization of TUG1 in Huh7 cells (Figure 3A). It revealed that the nucleus was the predominant location of TUG1, indicating likely involvement of TUG1 in the control of nuclear gene expression. Control of ROMO1 via the USF1 transcription factor was projected as the likely TUG1 mechanism of action by using the LncMAP database (bio‐bigdata.hrbmu.edu.cn/LncMAP/) (Figure S1A).^27^ ROMO1 expression was found in the GEPIA2 database (gepia.cancer‐pku.cn) (Figure S1B).^28^ In the TIMER database (cistrome.org/TIMER) (Figure S1C), relationships between the expression of USF1 and TUG1, and USF1 and ROMO1 were detected.^29^ UALCAN's gene expression dataset (ualcan.path.uab.edu) showed that the expression of USF1 in tumor tissues was significantly higher relative to normal tissues (Figure 3B). USF1 expression in HCC tumor tissues, Huh7, and L02 cells was measured by Western blotting (Figure 3C,3D). The findings revealed that USF1 was strongly expressed in Huh7 cells and tumor tissues of HCC relative to their respective controls. In addition, the findings demonstrated positive associations between ROMO1 and lncRNA TUG1 expression in HCC tissues (Figure 3E).

The dual‐luciferase reporter assay further confirmed the impact of lncRNA TUG1 on the promoter function of ROMO1. The findings revealed that the OE‐TUG1‐transfected cells displayed greater ROMO1 promoter function than those transfected with OE‐NC. In response to sh‐TUG1 transfection, reduced ROMO1 promoter activity was seen compared to sh‐NC transfection, suggesting that lncRNA TUG1 may directly control ROMO1 gene expression (Figure 3F). Deeper exploration of the process by which lncRNA TUG1 controlled ROMO1 gene expression was facilitated by a RIP assay that examined whether the USF1 transcription factor could interact with lncRNA TUG1 (Figure 3G). In contrast to IgG, the USF1 interaction with lncRNA TUG1 was enhanced, suggesting direct binding between the two. JASPAR and UCSC were used to determine the location of USF1 protein binding to ROMO1 DNA, which was predicted to contain two regions of attachment to the ROMO1 promoter (Figure 3H). The luciferase reporter gene assay (Figure 3I,3J) revealed site 1 (‐1582) to be the unique binding position. A ChIP assay was then used in Huh7 cells to investigate interactions between USF1 and site 1 (Figure 3K). More amplification products were obtained from site 1 primers than distal primers or the IgG control. Importantly, neither set of primers displayed any amplification products from protein lysates immunoprecipitated with an IgG control antibody, nor did they show any variation. This confirmed that the amplification products were specifically in the ROMO1 promoter region's site 1 (CATGTGT). We then silenced lncRNA TUG1 in Huh7 cells used for ChIP assay to further assess its function (Figure 3L). Fewer ROMO1 site 1 primer qRT‐PCR amplification products were seen from sh‐TUG1 transfected cells than sh‐NC transfected cells in lysates immunoprecipitated using the USF1 antibody. Subsequently, transfection with sh‐NC+OE‐NC, sh‐NC+OE‐TUG1, and sh‐USF1+OE‐TUG1 was performed to confirm that lncRNA TUG1 controlled ROMO1 expression by interacting with USF1. In response to shNC+OE‐TUG1 transfection, the amount of ROMO1 protein was significantly increased compared to cells transfected with sh‐NC+OE‐NC. Importantly, levels of USF1 and ROMO1 declined more following sh‐USF1+OE‐TUG1 transfection than sh‐NC+OE‐TUG1 transfection, (Figure 3M), while USF1 amounts stayed the same, suggesting that ROMO1 expression was modulated by lncRNA TUG1 through USF1 recruitment.

### 3.4 HCC cell growth, motility, and metastasis were enhanced by upregulated lncRNA TUG1 through USF1‐dependent ROMO1 overexpression

**FIGURE 4:**
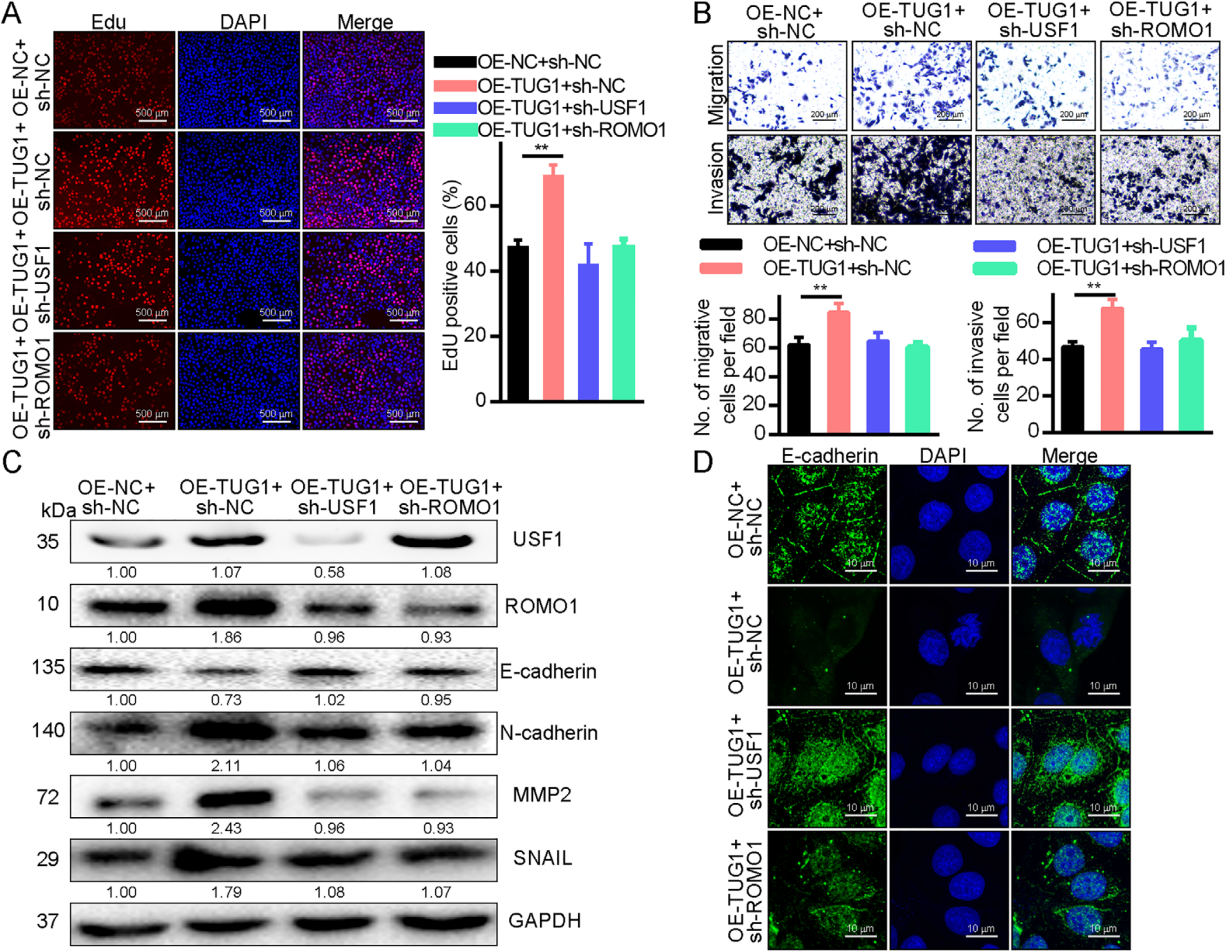
Growth, motility, and metastasis of HCC cells are modulated by overexpression of ROMO1 caused by upregulated lncRNA TUG1 through USF1 transcription factor mobilization. A, EdU staining for HCC cell propagation determination (×40), **P < .01. B, The capacity of HCC cells to move and invade was examined via a Transwell assay (×100), **P < .01. C, GAPDH was used as a Western blotting loading control when HCC cells were assayed for E‐cadherin, MMP‐2, ROMO1, SNAIL, N‐cadherin, and USF1 expression. D, Immunofluorescence was used to determine the E‐cadherin levels. Data are shown as mean ± standard deviation. Tukey's post hoc test after one‐way ANOVA compared multiple groups. Three independent experimental replicates were performed

The impact of lncRNA TUG1 on HCC cell physiological properties was examined by measuring cell growth, motility, and metastasis in Huh7 cells transfected with OE‐TUG1 + sh‐NC, OE‐NC + sh‐NC, OE‐TUG1 + sh‐ROMO1, and OE‐TUG1 + sh‐USF1. Following OE‐TUG1 transfection, the EdU staining of Huh7 cells (Figure 4A) indicated increased proliferation while proliferation declined following sh‐ROMO1 or OE‐TUG1 + shUSF1 transfection. Cell invasion and motility were then examined with a Transwell assay (Figure 4B). HCC cell invasiveness and motility both increased following OE‐TUG1 transfection. Transfection with sh‐ROMO1 or OE‐TUG1 + sh‐USF1 decreased invasiveness and motility. Figure 4C shows the results of Western blotting which revealed that OE‐TUG1 transfection raised MMP‐2, ROMO1, SNAIL, and N‐cadherin expression while reducing that of E‐cadherin. These results were reversed by transfection of either sh‐USF1 or sh‐ROMO1. Immunofluorescence confirmed these results (Figure 4D). Moreover, silencing ROMO1 did not significantly affect USF1, while silencing USF1 greatly diminished USF1 levels. Importantly, silencing ROMO1 blocked cell growth, motility, and metastasis that was enhanced by upregulating USF1 and lncRNA TUG1 expression (Figure S2). Hence, recruitment of the USF1 transcription factor to promote ROMO1 expression thereby triggering HCC cell growth, motility, and metastasis follows raised lncRNA TUG1 expression.

### 3.5 TUG1 silencing inhibits in vivo HCC development

**FIGURE 5:**
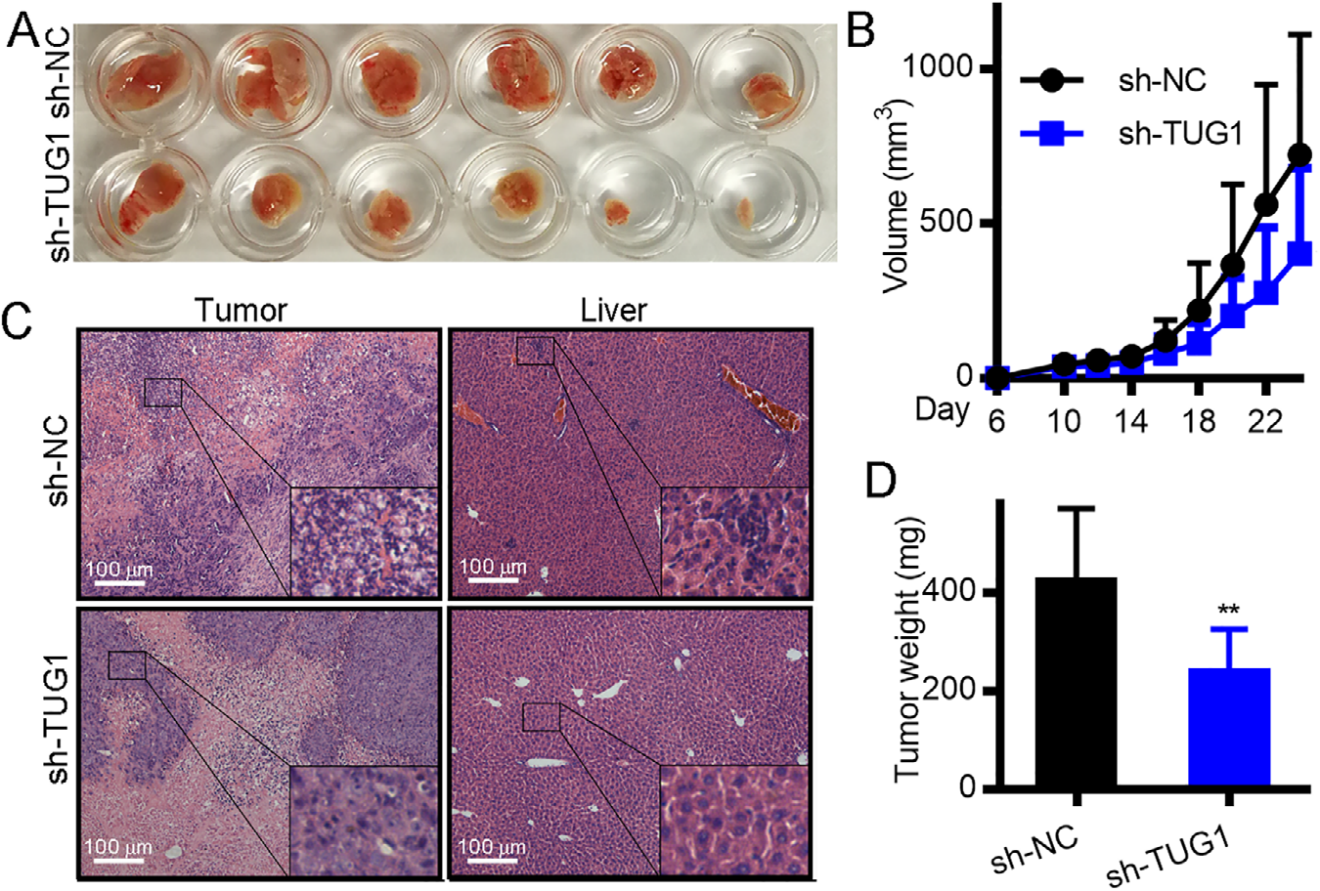
Silencing of TUG1 suppresses HCC tumor development. A, Typical images of tumors. B, The growth curve of each cohort of transplanted tumors. C, H&E‐detection of HCC cell infiltration in liver sections and tumor transplants (×100). D, Transplanted tumor weight for each group, **P < .01. Results are presented as means ± standard deviation and were compared using unpaired *t*‐tests. ANOVA compared data between multiple cohorts using the Bonferroni adjustment

Transfection of cells with sh‐TUG1 and sh‐NC lentiviruses was carried out to further explore the impact of TUG1 on HCC tumor growth by injecting transformed cells to develop in vivo tumors in the mice. Images of the transplanted tumors are shown in Figure 5A. Tumor dimensions, including mass and volume, are shown in Figure 5B.

For each mouse cohort, H&E staining identified metastatic cells (Figure 5C). The findings showed that mice with sh‐TUG1‐transfected tumors had lower ratios of liver invasion than those transfected with sh‐NC. The xenograft weight and average volume declined in sh‐TUG1‐transformed mouse tumors relative to sh‐NC‐transformed tumors (Figure 5D). Hence, silencing ROMO1 inhibited growth and liver metastasis of tumors stimulated by the joint upregulation of USF1 and lncRNA TUG1 (Figure S3). Taken together, the overall results described above demonstrate that TUG1 silencing blocked in vivo HCC pathogenesis.

## 4 DISCUSSION

**FIGURE 6:**
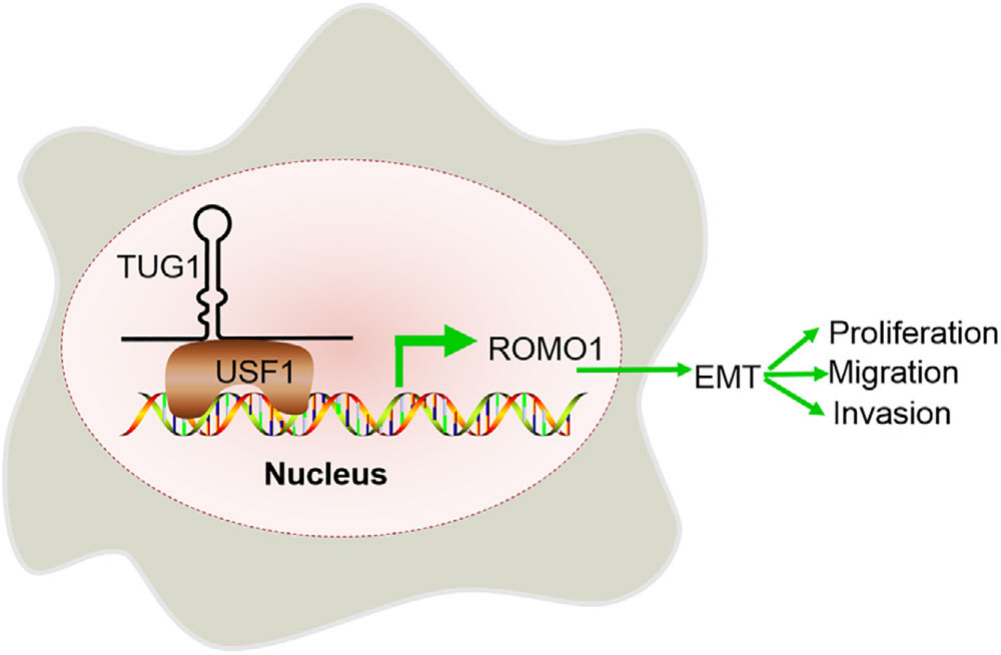
An illustration of lncRNA TUG1's practical significance to HCC. Cellular levels of ROMO1 are raised by lncRNA TUG1 through mobilization of the USF1 transcription factor to facilitate the growth of HCC. This is demonstrated by increased growth, motility, and metastasis of HCC cells

Usually, HCC is characterized by aggressive metastasis and a high propensity for propagation, leading to poor prognosis, likely resurgence, and extreme tissue penetrance.^30^ An appreciation of the different molecular processes that underpin HCC development may facilitate rapid detection and better treatment. New evidence into HCC carcinogenesis and proliferation indicates the critical functions of lncRNAs^31^; thus, it is crucial to explore lncRNAs as potentially new HCC treatment targets and biomarkers. In the present investigation, we analyzed the role of lncRNA TUG1 in HCC in the USF1/ROMO1 complex (Figure 6).

LncRNAs influence various cancers such as HCC by affecting crucial activities including cell growth, motility, and metastasis.^19^, ^32^ Our results show that undesirable HCC prognostic outcomes are associated with elevated lncRNA TUG1 expression. Microscopic HCC cell classifications correlated with abnormal lncRNA expression features and were used to determine prognosis.^26^, ^33^, ^34^ Moreover, the survival and staging of prostate and colorectal cancer have been found to be associated with lncRNA TUG1, demonstrating its potential for cancer prognosis.^34^, ^35^ Although there is a lack of published work regarding the functions of lncRNA TUG1 in HCC, its influence on cytohistological processes has been reported, further reinforcing our findings. It is noteworthy that lncRNA TUG1 is a major regulator of cell apoptosis and inflammation.^36^ Our experiments with and without the use of animals revealed that blocking nude mice tumor development occurred in tandem with decreased HCC cell motility, metastasis, and proliferation associated with lncRNA TUG1 silencing. LncRNA TUG1 is known to mediate multiple processes. Regulation of the TWIST transcription factor was previously shown through mechanistic experiments to be the mechanism through which lncRNA TUG1 controlled malignant cell propagation and motility in colorectal cancer.^35^ In a separate study, lncRNA TUG1 was shown to elevate sensitivity to cytokines by blocking the inhibition of phosphate and tensin homolog (PTEN) through microRNA‐221 in non‐small cell lung cancer.^37^ In this study, we have shown that ROMO1 overexpression mediated by USF1 transcription factor mobilization and, in turn, mediated by lncRNA TUG1 stimulated HCC cell motility, metastasis, and proliferation.

The transcription factor USF1 is a recognized breast cancer oncogene that can promote Epithelial‐mesenchymal transition (EMT) via transforming growth factor β1 (TGF‐β1) overexpression leading to stimulation of TGF‐β1/Smad signaling.^38^ It has also been shown to induce mucin 13 transcriptions to encourage glioblastoma stem cell proliferation.^39^ We found ROMO1 transcription to be activated in HCC cells and tissues due to their expression of large quantities of USF1. There was a negative correlation between the survival of HCC cases and ROMO1 upregulation. Decreased cell specialization, greater tumor volume, and tumor penetration of blood vessels all correlated with large amounts of ROMO1.^24^ ROMO1 is therefore considered an attractive cancer treatment target.^40^ LncRNA potentially controls a broad array of cell properties due to its ability to use USF1 to activate ROMO1. In particular, our present findings support USF1 transcription factor‐mediated activation of ROMO1 expression by lncRNA TUG1 to aggravate HCC cell motility, growth, and metastasis.

## 5 CONCLUSION

The information presented from our research strengthens the view that the lncRNA TUG1/USF1/ROMO1 complex is involved in HCC's metastatic capacity and can be targeted for therapeutic deployment. Our key finding is that lncRNA TUG1 upregulation promotes HCC growth by attracting the USF1 transcription factor to raise ROMO1 expression. Based on these results, additional research and exploration of large biochemical databases to uncover the specific inherent pathways involved are justified.

## AUTHOR CONTRIBUTIONS

Study concept and design: Liu, He, and Pan. Data collection: Qiu, He, and Liu. Statistical analysis and preparation of the figures: Cai and Liu. Drafting of the manuscript: Tian. Critical revision of the manuscript for important intellectual content: Liu, Qiu, Guifang He, and Weitai He. All authors read and approved the final manuscript.

## AVAILABILITY OF DATA AND MATERIALS

The datasets generated and/or analyzed during the current study are available from the corresponding author on reasonable request.

## ETHICS APPROVAL AND CONSENT TO PARTICIPATE

The study was conducted with the approval of the Ethics Committee of the Affiliated Hospital of Qingdao University. All participating patients provided written informed consent. Nude mice used for in vivo experimental animal studies were cared for in accordance with a protocol approved by the Laboratory Animal Care and Use Committee of the Affiliated Hospital of Qingdao University (AHQU20180822A).

## CONFLICT OF INTERESTS

The authors declare that there is no conflict of interest that could be perceived as prejudicing the impartiality of the research reported.

## Supporting information

SUPPORTING INFORMATION
