# ERRATUM

**DOI:** 10.1002/mco2.70

**Published:** 2021-04-29

In Pan et al.,^1^ Figure S2 was incorrectly published as Figure 2.

The correct Figure 2 is below:

**FIGURE 2:**
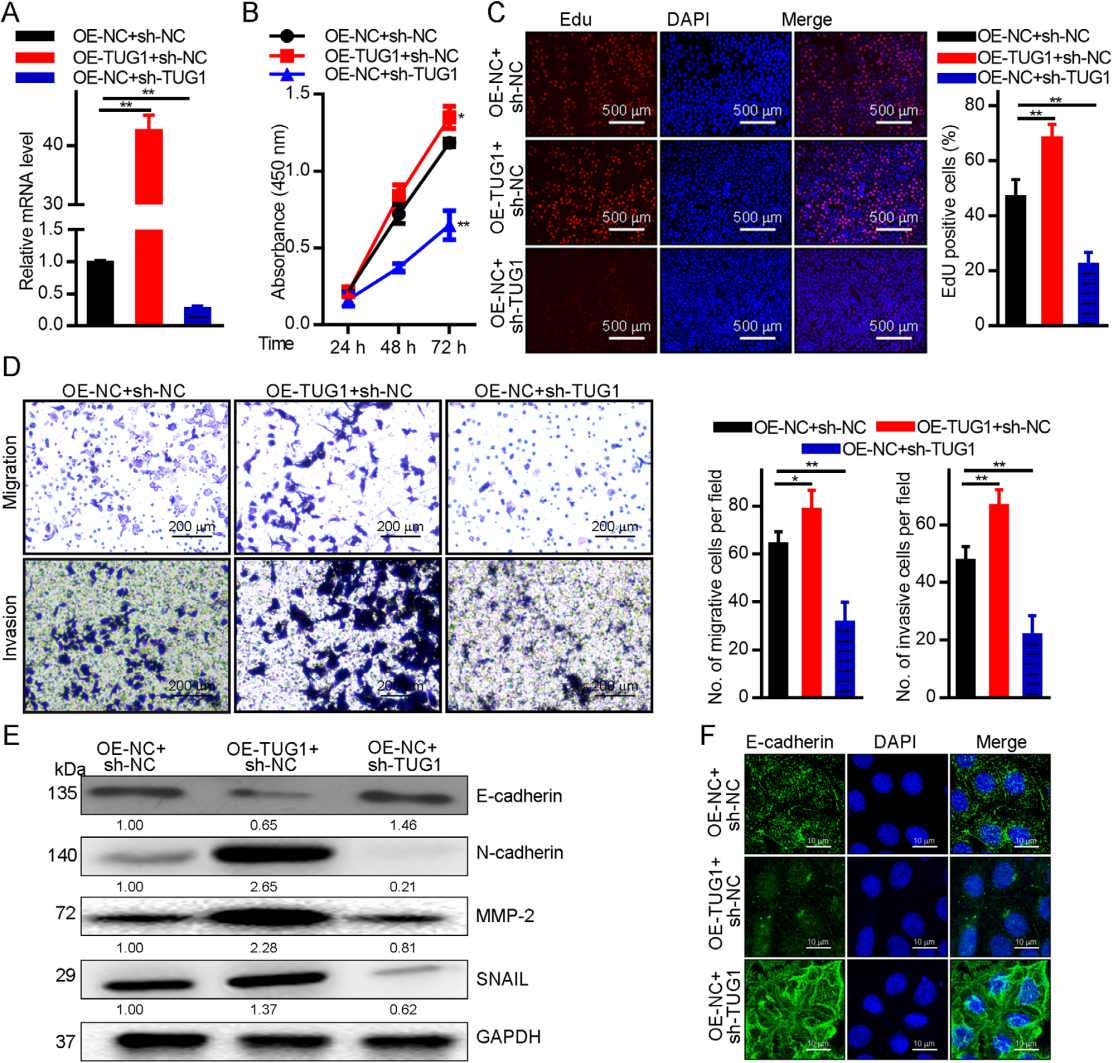


The online version of this article has been corrected.

The publisher regrets this error.
