# Supplementary material for: TUG1 long non‐coding RNA enlists the USF1 transcription factor to overexpress ROMO1 leading to hepatocellular carcinoma growth and metastasis
Source: MedComm (2020). 2020 Nov 26;1(3):386–99. doi: 10.1002/mco2.38 (PMC8491240; doi:10.1002/mco2.38)
Supplement: Supplementary file 1 — SUPPORTING INFORMATION [file MCO2-1-386-s001.docx]

# Supplementary

Supplementary Table 1: Sequences of primers

| Name | Sequences (5′-3′) |
| --- | --- |
| TUG1-RT-F | GAACTACTGCGGAACCTCAA |
| TUG1-RT-R | ACTTGGTGAGCACCACTCC |
| USF1-RT-F | ATGCTGGATACTGGACACA |
| USF1-RT-R | AAAGGAAGAACCAATGGAAGT |
| ROMO1-RT-F | AAGCTGCTTCGACCGTGTC |
| ROMO1-RT-R | CCCGCATTCCGATCCTGAG |
| GAPDH-RT-F | GGCATGGACTGTGGTCATGAG |
| GAPDH-RT-R | TCATGGGTGTGAACCATGAGAA |
| ROMO1-P1-F | GGGGTACCAGAACTATTGAGCTCTTACTAT |
| ROMO1-P1-R | CCCTCGAGGTGGGCTCTGCCAGCGGGACCAT |
| ROMO1-P2-R | CCCTCGAGACTCACTAGCAATTGACCTTGGT |
| ROMO1-P3-R | CCCTCGAGAGGCAGCCATGGAACGTGCTCGT |
| ROMO1-MUT1-F | TCTCCCAGCCATAAGTTTATCTCCGGGAGTGTGGGTT |
| ROMO1-MUT1-R | AACCCACACTCCCGGAGATAAACTTATGGCTGGGAGA |
| ROMO1-MUT2-F | GGTAACGGTCTGTTTATCTGCCCGGAGCAGCATCTGT |
| ROMO1-MUT2-R | ACAGATGCTGCTCCGGGCAGATAAACAGACCGTTACC |

## Supplementary Figure


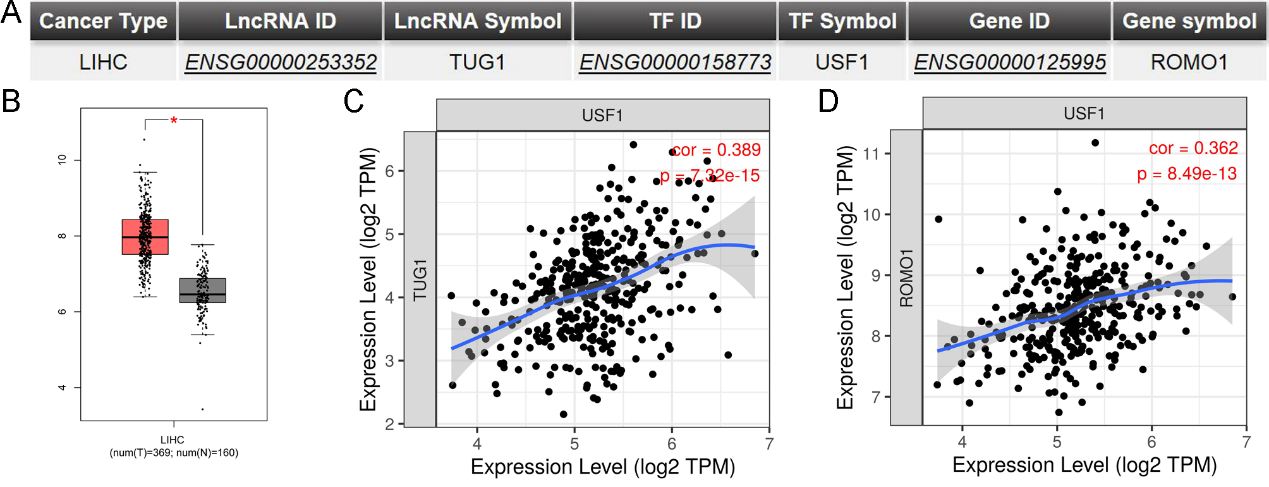


## Figure S1

Results of bioinformatics predictions. (A) Bioinformatics predictions of lncRNA TUG1 through LncMAP database. (B) The expression of ROMO1 was detected by GEPIA2. **p*<0.05. (C) The relationship between TUG1 and USF1 was detected by TIMER database. (D) The relationship between USF1 and ROMO1 was detected by TIMER database.


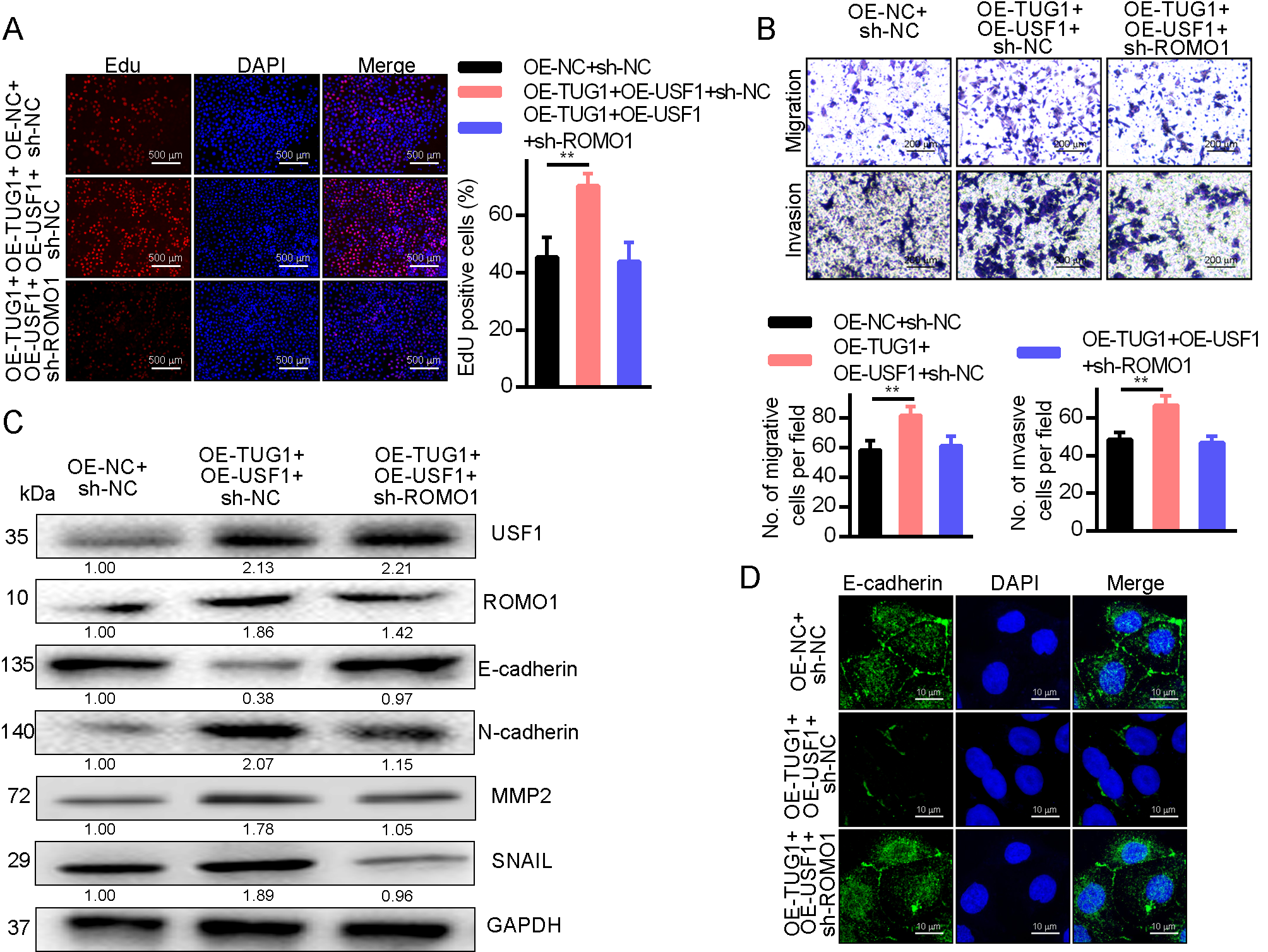


## Figure S2

LncRNA TUG1 affects HCC cell proliferation, migration and invasion by regulating ROMO1 expression. (A) EdU staining assay to examine the proliferation ability of HCC cells. Bar=500 μm. ***p*<0.01. (B) Transwell assay to examine the migration and invasion ability of HCC cells. Bar=200 μm. ***p*<0.01, (C) Western blotting analysis for expression of USF1, ROMO1, E-Cadherin, N-Cadherin, MMP-2 and SNAIL normalized to GAPDH in HCC cells treated with the OE-NC + sh-NC group, the OE-TUG1 + OE-ETS1 + sh-NC group or OE-TUG1+OE-USF1+shROMO1. (D) The E-cadherin protein levels were detected by immunofluorescence. Cell experiment was repeated three times independently.


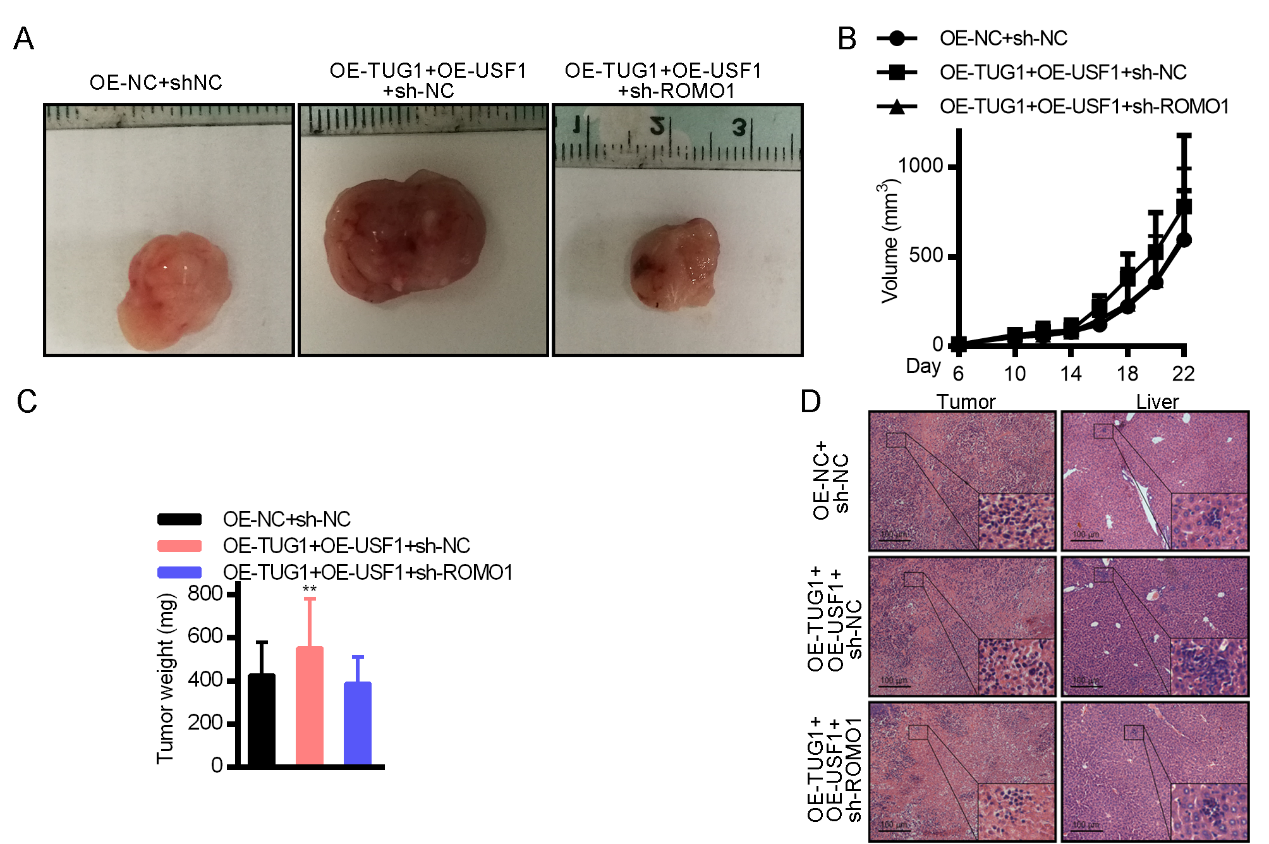


## Figure S3

LncRNA TUG1/ETS1/ROMO1 axis affects the development of HCC *in vivo*. A) Representative images of xenograft tumors. B) Representative volume quantitation of xenograft tumors. C) The weight quantitation of xenograft tumors, ***p*<0.01. D) The invasion of HCC cells in transplanted tumor and liver sections detected by H&E (100 ×). The values in the figures were quantitative data, n = 6. Quantitative data were expressed as means ± standard deviation. Comparisons between time-based measurements were performed with repeated measures ANOVA.
